# Shear wave elastography combined with electromyography to assess the effect of botulinum toxin on spastic dystonia following stroke: A pilot study

**DOI:** 10.3389/fneur.2022.980746

**Published:** 2022-10-10

**Authors:** William Campanella, Angelo Corazza, Luca Puce, Laura Privitera, Riccardo Pedrini, Laura Mori, Leonardo Boccuni, Giovanni Turtulici, Carlo Trompetto, Lucio Marinelli

**Affiliations:** ^1^Department of Neuroscience, Rehabilitation, Ophthalmology, Genetics, Maternal and Child Health, University of Genova, Genova, Italy; ^2^IRCCS Ospedale Policlinico San Martino, Department of Neuroscience, Division of Neurorehabilitation, Genova, Italy; ^3^Unità di Radiologia Diagnostica ed Interventistica Istituto Ortopedico Galeazzi di Milano, Milan, Italy; ^4^Institut Guttmann, Institut Universitari de Neurorehabilitació adscrit a la UAB, Barcelona, Spain; ^5^Universitat Autònoma de Barcelona, Cerdanyola del Vallès, Spain; ^6^Fundació Institut d'Investigació en Ciències de la Salut Germans Trias i Pujol, Barcelona, Spain; ^7^S.C. Radiodiagnostica Ospedale Evangelico Internazionale di Genova, Genova, Italy; ^8^IRCCS Ospedale Policlinico San Martino, Department of Neuroscience, Division of Clinical Neurophysiology, Genova, Italy

**Keywords:** shear wave, elastosonography, spasticity, muscle tone, muscle hypertonia, stiffness

## Abstract

**Background:**

Shear wave elastography (SWE) is a method for carrying out a quantitative assessment of the mechanical properties of soft tissues in terms of stiffness. In stroke survivors, the paretic muscles may develop hypertonia due to both neural-mediated mechanisms and structural alterations with consequent muscular fibrous-fatty remodeling.

**Methods:**

Fourteen adult patients with spastic dystonia following stroke were recruited. Muscle hypertonia was assessed using the modified Ashworth scale (MAS). Muscle activation was measured by surface electromyography (sEMG) with the selected muscle in shortened (spastic dystonia) and stretched (dynamic stretch reflex) positions. SWE was performed on a selected paretic muscle and on the contralateral non-paretic one to calculate shear wave velocities (SWV) along and across muscular fibers. The modified Heckmatt scale (MHS) pattern was also determined. All evaluations were performed shortly before BoNT-A injections (T0) and one month later (T1).

**Results:**

All SWV on paretic muscles were higher than contralateral non-paretic ones (*p* < 0.01). After BoNT-A injection, a significant reduction in MAS (*p* = 0.0018), spastic dystonia (*p* = 0.0043), and longitudinal SWE measurements, both in shortened (*p* = 0.001) and in stretched muscular conditions (*p* = 0.0029), was observed. No significant changes in SWV on non-paretic muscles were observed. Higher SWV resulted along the direction of muscular fibers vs. across them (*p* = 0.001). No changes resulted from the MHS evaluations after BoNT-A. There was a positive correlation between MHS scores and SWV values while the muscle was in the shortened position, but not with spastic dystonia recorded by sEMG.

**Conclusions:**

This is the first study evaluating the effect of BoNT-A on muscle hypertonia following stroke, assessed by both SWE and sEMG. These findings support SWE as a useful method to disclose intrinsic muscular remodeling, independently of the effect of spastic dystonia, in particular, while muscles were assessed in a neutral position. SWE measurements of muscle stiffness cannot tell apart neural-mediated and intrinsic muscle hypertonia. Interestingly, when sEMG activity is very limited, as in spastic muscles kept in a shortened position, SWE can provide a measurement of stiffness due almost completely to intrinsic muscle changes. Alongside sEMG, SWE could aid clinicians in the assessment of responses to treatments.

## Introduction

Following a stroke, paretic limbs often develop muscle hypertonia within the so-called upper motor neuron syndrome. Spasticity is the most frequently investigated cause of increased muscle tone, occurring in about 38% of post-stroke patients (1). Spasticity can be clinically appreciated as a velocity-dependent increase in muscle tone during passive stretching, due to increased excitability of the stretch reflex resulting from upper motor neuron lesions (2, 3). Spastic dystonia is less commonly evaluated, but recent studies suggest that it may occur more frequently than spasticity (4). Like in muscles with spasticity, those with spastic dystonia show a stretch reflex during passive muscle lengthening, but, differently from spasticity, electromyographic activity reflecting muscle activation is present also at rest, with the muscle in a neutral or shortened position, despite relaxation attempts (2). Spastic dystonia probably shares the same neural substrate as spasticity, but likely contributes more importantly to muscle shortening and disability (5, 6). Surface electromyography (sEMG) is considered the gold standard to detect electric muscle activation and is therefore ideal to measure spasticity and spastic dystonia, which represent the main contributors to muscle tone increase in post-stroke patients. Importantly, reduced limb mobility affects muscle structure: sarcomere loss, an increase of connective tissue, and muscle contracture produce intrinsic hypertonia that sums up with spasticity and spastic dystonia but cannot be directly assessed by sEMG.

Clinicians should discriminate with greater precision the increase in muscle tone after stroke, distinguishing the neuro-mediated reflex hypertonia (from now on called “reflex hypertonia”) from the intrinsic one, to define the correct treatment and assess the therapeutic impact. The modified Ashworth scale (MAS) is the most feasible measurement of spasticity in clinical practice (7). However, the MAS is based on a subjective and non-quantitative assessment and this restricts its application. Moreover, considering the validity, reliability, and sensitivity, MAS is not considered an ideal scale for the assessment of muscle hypertonia (8).

Conventional B-mode ultrasonography (US) is widely utilized for musculoskeletal pathology as a first-line approach mainly because of real-time access and relatively low costs (9). Sonoelastography can assess the mechanical properties of soft tissues by US imaging. In particular, a further development of such technique, called “shear wave elastography” (SWE), provides a quantitative evaluation of elastic properties by measuring the propagation velocity distribution of the directional shear waves, produced by an ultrasound pulse (10).

In the recent years, SWE has been introduced as a promising diagnostic tool in musculoskeletal pathology to assess muscle and tendon elastic properties related to degeneration, injury, and healing (11, 12). Recently some authors demonstrated good to excellent inter-observer reproducibility and intra-observer repeatability in radiologists, validating the feasibility and reliability of SWE to assess mechanical properties of skeletal muscle tissue in the upper and lower extremities (12–14).

In recent years, many studies (15–24) used SWE with different approaches and protocols, to quantify muscular stiffness related to spasticity.

Shear wave velocity (SWV) increases with increasing tissue stiffness and is therefore influenced either by muscle fibrosis (intrinsic hypertonia) or muscle contraction (active or reflex). Compared to healthy muscles, paretic muscles therefore should demonstrate increased stiffness at rest (mostly because of intrinsic hypertonia, particularly if the muscle is in a shortened position) and even more while stretched or kept elongated (intrinsic hypertonia + reflex hypertonia) (6). An increase of SWV is therefore expected in resting paretic muscles compared to the healthy ones as well as in stretched compared to shortened conditions. Botulinum toxin should only reduce stiffness due to reflex hypertonia (recordable with sEMG), but not intrinsic hypertonia, as it is ineffective on fibro-adipose degeneration of the muscle.

In this context, SWE could gain an important role due to its potential capability to quantitatively assess the contribution of viscoelastic properties to soft tissue stiffness. The opportunity to match the sEMG evaluation of spastic dystonia before and after botulinum neurotoxin type A (BoNT-A) treatment, paralleled with SWE evaluation, can provide unprecedented information about the contribution of reflex hypertonia to SWV.

Based on these assumptions, in this pilot study, the main objective was to assess the effect of botulinum toxin on post-stroke patients with spastic dystonia using both sEMG and SWE to confirm that muscle hypertonia improvement can be reflected by the reduction of both muscle electromyographic activity and SWV. Furthermore, we aimed to investigate the capability of SWE to estimate muscular changes in terms of elasticity in paretic limbs, due to the contribution of reflex hypertonia to overall stiffness.

## Materials and methods

### Population

From September 2018 to December 2019, we recruited 14 patients (5 females) with a median age of 61 years (range 42–78). Inclusion criteria were as follows: (1) presence of spastic dystonia (presence of EMG activity at rest that cannot be voluntarily silenced by the subject) (3) in at least one muscle group (e.g., elbow flexors) of only one side of the body due to ischaemic or hemorrhagic stroke occurred at least 3 months before, (2) unaffected contralateral body side, (3) for patients treated with botulinum toxin, at least 4 months must have passed since last injection, (4) age of 18 years or more, and (5) ability to understand and sign the informed consent. Exclusion criteria were concomitant parkinsonism and history of lesions affecting the muscle to be evaluated. The institutional review board at IRCCS Ospedale Policlinico San Martino (Genoa, Italy) approved the present study and all patients provided written informed consent before data collection and assessment.

### Assessment

All enrolled patients underwent a neurological examination and assessment of muscle hypertonia applying the MAS. Among those muscles where botulinum toxin injection was indicated, a muscle with MAS > 0 was selected for the consequent sEMG and SWE assessments. Muscles characterized by large bulk and reduced distance from the ultrasound transducer (such as biceps brachii muscle) were preferred.

All patients underwent sEMG, conventional B-mode US for the qualitative identification of muscle echogenicity applying the modified Heckmatt Scale (MHS) and the quantitative evaluation of SWV, reflecting muscle stiffness, through SWE ultrasound evaluation. The latter was also performed at the contralateral homolog non-paretic muscle. The ultrasound evaluations were performed by an experienced radiologist (Corazza). The examiners evaluated the patients independently and were blind to each other.

All MAS, sEMG, MHS, and SWV evaluations were carried out just before the injection of BoNT-A (T0) and then repeated 4 weeks (range: 22–32 days) later (T1) according to the same protocols: this time interval was chosen to intercept the time when the maximum effect of BoNT-A is expected after injection.

### Electromyography

Self-adhesive electrodes (Neuroline 700, Ambu A/S, Ballerup, Denmark) were placed 1 cm apart over the muscle belly for bipolar recording following SENIAM (Surface Electromyography for Non-Invasive Assessment of Muscles) guidelines (25). Signals were acquired by a BIOAMP LT unit (Vertigo, Genoa, Italy) and underwent a 20 to 500 Hz band-pass filter for offline processing using custom software running in Labview (National Instrument, Austin TX, USA). Subjects were lying supine on the examination table in a quiet room while attempting to remain relaxed (the prone position has been preferred in the case of triceps surae muscle).

Spastic dystonia was assessed by recording sEMG, while the muscle remained in a neutral position, before performing passive limb movements. Each subject entered the analysis with the average rectified value (ARV) during 30 s of recording. A dynamic stretch reflex was also assessed in the selected muscle by recording sEMG during 10 passive stretches performed at a reproducible speed, according to a previously validated methodology (26). The selected muscle was stretched with linear (ramp and hold) passive movements following the pacing provided by an emulated metronome. The beats per minute pace (BPM) was decided for each patient according to the muscle and the amount of hypertonia and maintained the same at both time points. The ARV was calculated for each movement only during the dynamic phase of muscle stretching. Each subject entered the analysis with the mean ARV obtained from 10 consecutive stretches before and after BoNT-A injections.

### Sonoelastography

A LOGIQ E9 ultrasound system equipped with a 9L linear array transducer with Shear Wave Elastography technology (GE Healthcare, General Electric Company, Chicago, Illinois, US) was used to acquire B-mode images and SWE measurements. Such technology uses a “comb-push” excitation source (multiple pushing beams transmitted on the transducer simultaneously in a comb-like pattern), time-interleaved shear wave tracking (data interpolation), directional filters, and a time-of-flight algorithm which is used to estimate the local SWV at every location in the region of interest.

**Figure 1:**
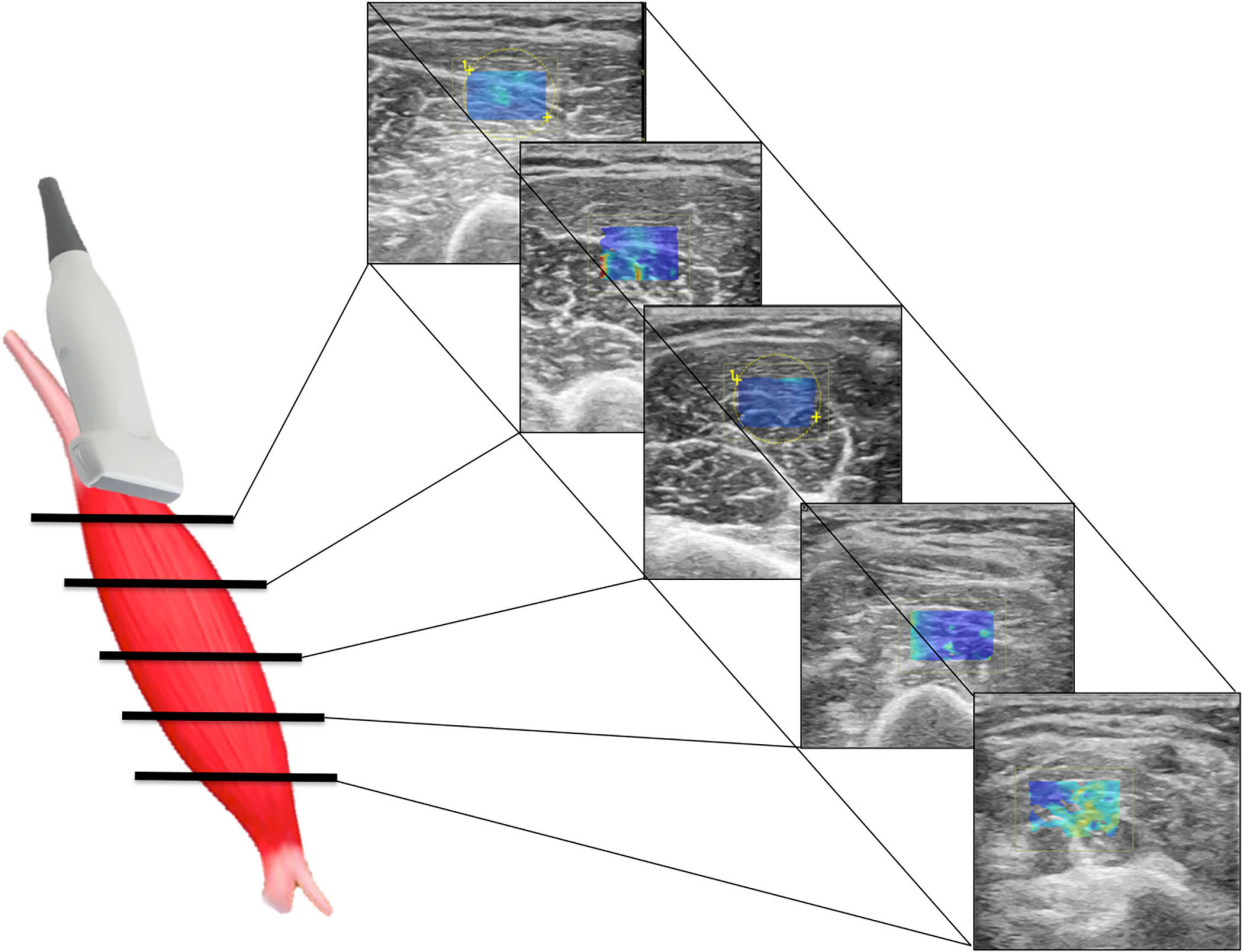
Scheme illustrating SWE acquisition on the short axis of muscular fibers: the transducer is placed on the muscle belly performing consecutive axial scans to assess the maximum amount of muscle (left); in each scan, SW velocities are measured in the positioned rectangular ROI generating the relative color-coded elastogram superimposed to the B-mode image (right). The stiffness at any location within the ROI is sampled using the measurement tool (dotted circles). A final median value is then expressed.

**Figure 2:**
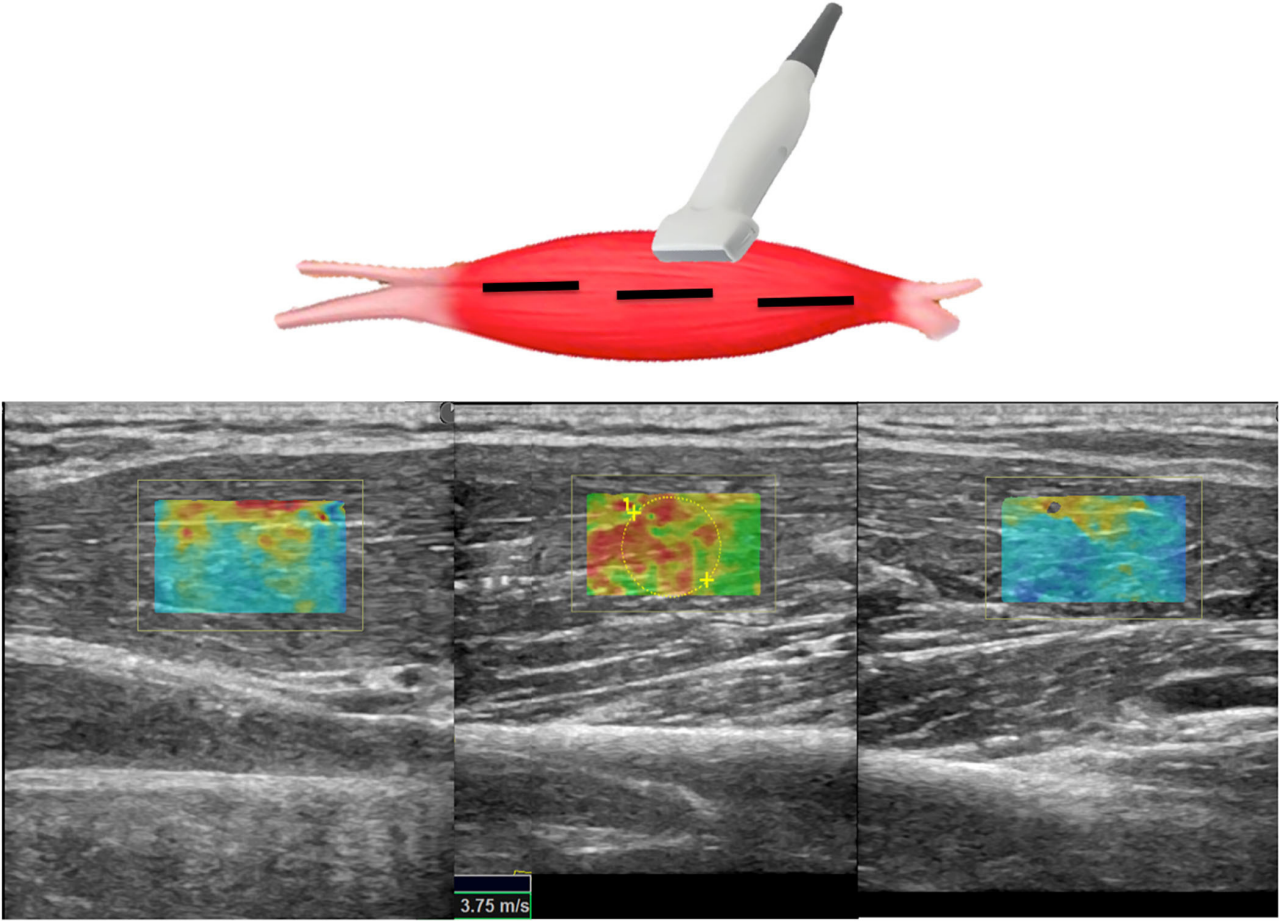
Scheme illustrating SWE acquisition on the long axis of muscular fibers: the transducer is placed on the muscle belly performing consecutive longitudinal scans to assess the maximum amount of muscle; in each scan, SW velocities are measured in the positioned rectangular ROI generating the relative color-coded elastogram superimposed to the B-mode image (below). The stiffness at any location within the ROI is sampled using the measurement tool (dotted circles). A final median value is then expressed.

Patients have been placed in the supine position; the paretic arm (P) of the subject was first held in a shortened (sh) position. Axial scans were obtained with the US probe positioned on the skin surface on the short axis (Ax) of the muscle being examined. Standard machine settings included a maximum image depth of 10 cm, scanning frequency of 7 MHz, single image focus, tissue harmonic imaging, speckle reduction function turned off, and setting meters per second (m/s) as the unit of measurement of the SWV (15). Particular attention was paid to applying the least possible pressure on the underlying tissues, to avoid any additional, albeit minimal, compression on the muscle by increasing the risk of obtaining overestimated measurements. The quantitative values of the SWV for muscle stiffness estimation were measured using a rectangular region of interest (ROI) with variable dimensions (maximum dimensions: 2 x 2 cm), set to a depth between 1 and 5 cm from the skin surface and displayed on the screen as an encoded color map of the SWVs detected. The objective of the procedure was to achieve reproducible SWV measurements, avoiding the detection of aponeurosis and/or thickest intramuscular septa within the ROI and by searching, at the same time, to cover the wider area of muscle fibers (27). Further, analogous SWE measurements were repeated by rotating the probe on longitudinal planes of the muscle (Lo) (Figures 1, 2). This procedure was repeated on the same muscle in a stretched condition (st), while a second operator was keeping the arm of the patient passively extended. Subsequently, the protocol was performed on the contralateral homolog non-paretic muscle (NP). For each condition, 10 measurements were collected and the median value entered consequent analyses.

### Modified Heckmatt scale

Meantime, since the SWE evaluation was provided based on a conventional B-Mode US image, an MHS pattern (28) of the selected muscle was assessed by the same operator following the different degrees of muscle echogenicity.

### Botulinum toxin a injections

In total, 100-unit vials of incobotulinumtoxinA (Merz Pharma, Frankfurt am Main, Germany) were diluted with 2 ml of preservative-free normal saline (50 U/ml). Muscles were injected according to the current guidelines based on muscle size and amount of hypertonia. Generally, each muscle was injected with 20 to 100 IU and each patient received no more than 400 IU. All BoNT-A injections were performed under US guidance.

### Statistical analysis

Wilcoxon signed-rank test was used for all comparisons: MAS and MHS (T0-T1), electromyographic ARV for spastic dystonia (T0-T1), and SWE measures (T0-T1, P-NP, Ax-Lo, sh-st). Spearman rank test was used to assess the correlation between the Heckmatt scale and SWV measurements at T0. Continuous variables have been reported using mean ± SD, while non-continuous variables have been reported as median (1st to 3rd quartiles). Statistical significance was set for *p* < 0.01. This is a pilot study: no preliminary data were available to estimate the sample size.

## Results

All 14 patients were evaluated on paretic and contralateral healthy selected muscles for a total of 28 muscles (Table 1). None reported discomfort or any side effect during or after sEMG and SWE assessments. One subject (#14) had a severe reduction in the range of motion of the paretic limbs (MAS=4). Given the very limited joint excursion, in this patient, dynamic stretch reflex recording and stretched SWV assessments were unfeasible.

**Table 1:** Baseline patients' characteristics and assessed muscle.

**Patient**	**Gender**	**Age (y)**	**Affected side of the body**	**Time since stroke (y)**	**Target muscle**	
1	M	49	Hemorrhagic/left	5	Flexor Digitorum Sup	
2	M	78	Ischemic/right	3	Brachialis	
3	M	46	Ischemic/right	7	Soleus	
4	F	48	Hemorrhagic/right	1	Gastrocnemii	
5	F	57	Ischemic/right	6	Biceps Brachii	
6	M	65	Hemorrhagic/right	3	Brachialis	
7	M	62	Ischemic/left	5	Biceps Brachii	
8	F	46	Ischemic/right	14	Brachialis	
9	M	71	Ischemic/right	8	Gastrocnemii	
10	F	42	Ischemic/right	3	Gastrocnemii	
11	M	62	Ischemic/right	4	Biceps Brachii	
12	M	62	Ischemic/left	4	Brachialis	
13	F	60	Ischemic/left	2	Soleus	
14	M	69	Ischemic/left	15	Biceps Brachii	

### Modified Ashworth scale

**Figure 3:**
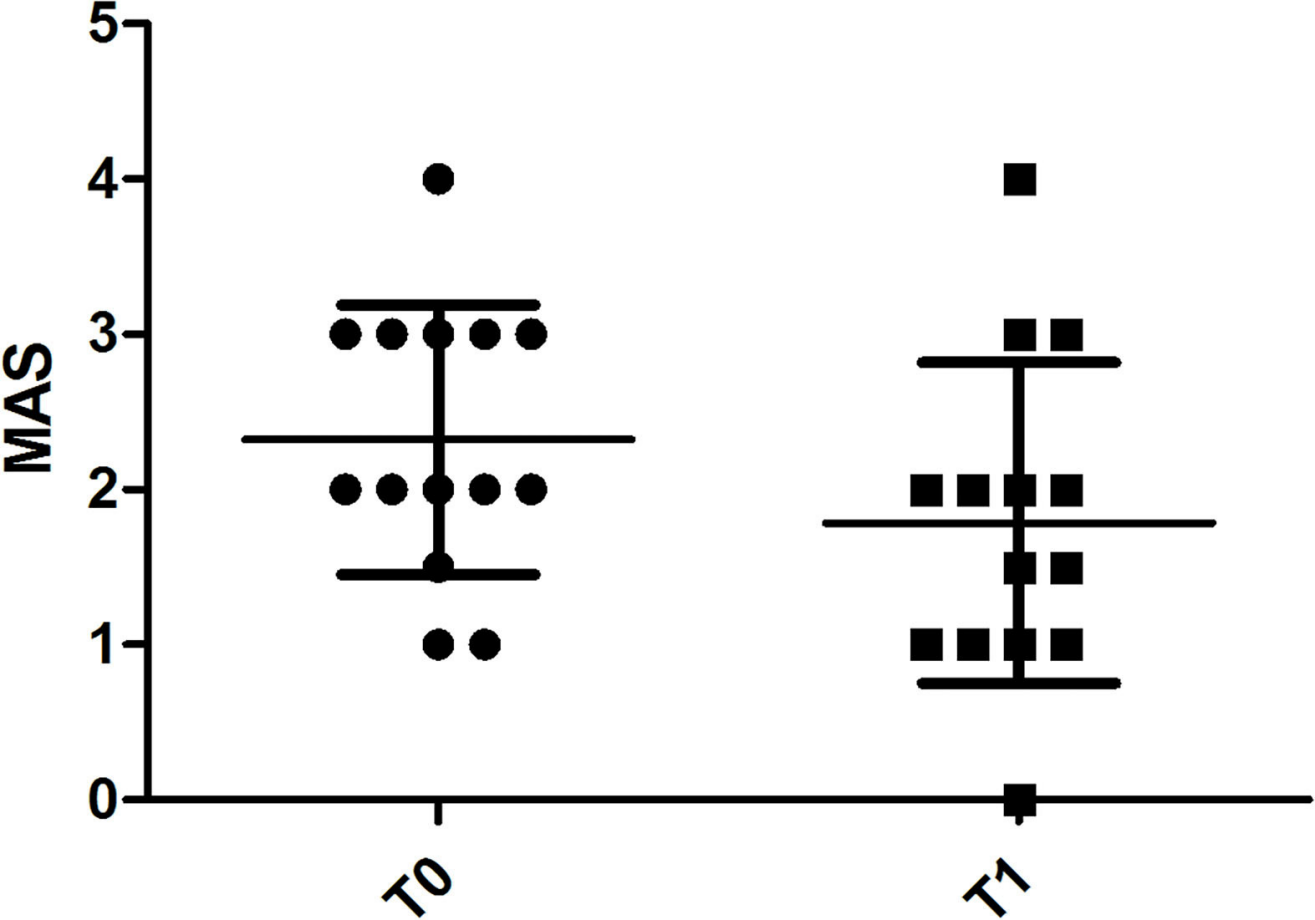
Chart illustrating the significant difference in MAS score assessments of spastic muscles before (T0) and 4 weeks after (T1) BoNT-A injection.

MAS values at T0 were 2 (2–3) and at T1 significantly decreased to 1.75 (1-2) (*p* = 0.018) (Figure 3).

### Surface electromyography

**Figure 4:**
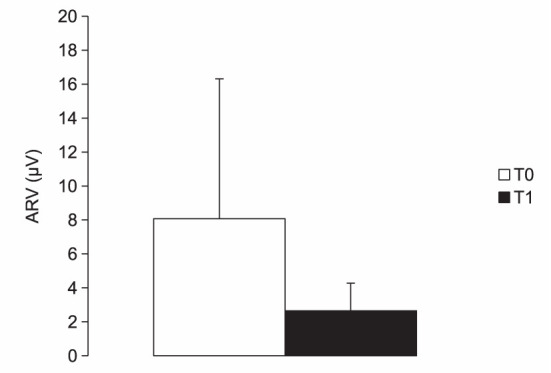
Chart illustrating the average rectified values (ARV) of electromyographic activity (μV) measured in muscles with spastic dystonia while kept in a shortened position before (T0) and 4 weeks after (T1) BoNT-A injection. See text for statistical comparison.

Spastic dystonia ARV was 8.07 ± 8.24 μV at T0 and 2.65 ± 1.62 μV at T1; dynamic stretch ARV was 18.13 ± 22.61 μV at T0 and 7.30 ± 3.70 μV at T1. Both spastic dystonia (*p* = 0.0043) and dynamic stretch (*p* = 0.0019) significantly decreased at T1 after BoNT-A injection (Figure 4).

### Shear wave elastography

A total of 224 SWV values resulted considering 14 patients evaluated in 16 conditions resulting from the combination of the four binary variables: time point (T0 vs. T1), body side (P: paretic limb vs. NP: non-paretic limb), transducer orientation (Ax: axial vs. Lo: longitudinal), and muscle condition (st: stretched vs. sh: shortened).

**Figure 5:**
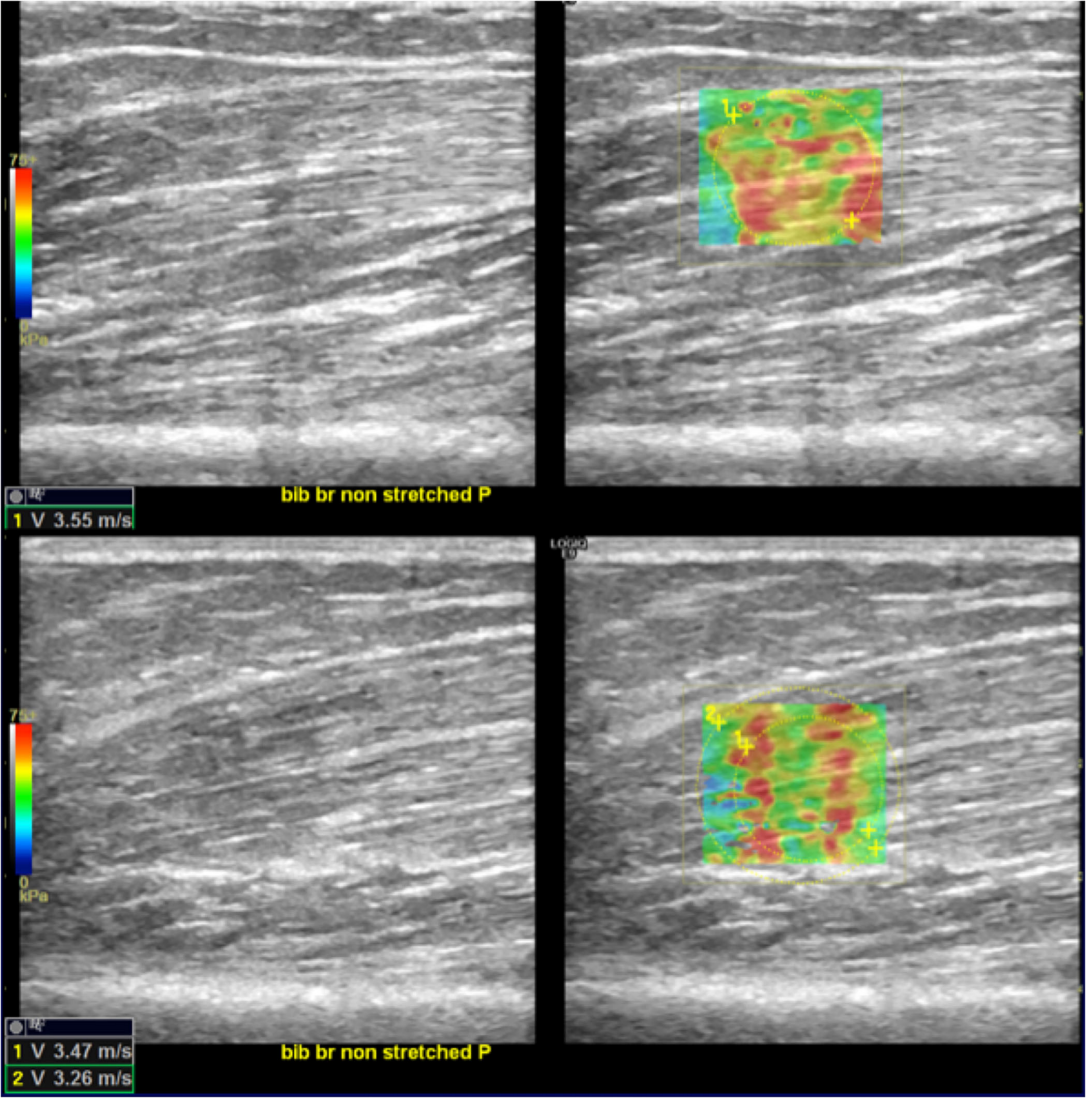
SWE reduction between T0 and T1. In the upper-right quadrant is illustrated an example of an elastogram (superimposed to the B-mode image) generated by an ROI placed in the biceps brachii muscular belly along the direction of the fibers at T0; in the lower right image, the same evaluation at T1 shows lower values (lower left corners of the images). Images on the upper- and lower-left quadrant are the respective B-mode conventional US image automatically paired on the screen by the SEL software.

**Figure 6:**
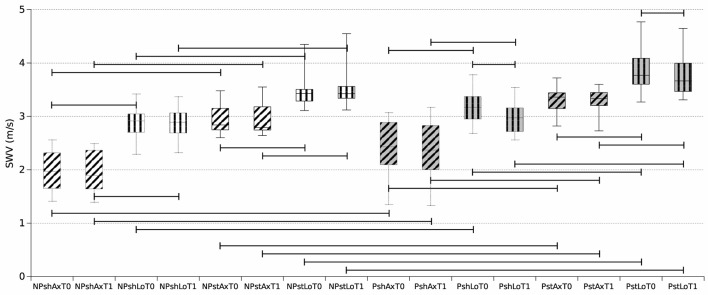
Boxplot reporting median and inter-quartile ranges of shear wave elastography velocity (SWV) values obtained during the 16 assessments. NP, non-paretic limb (white background); P, paretic limb (gray background); sh, shortened muscle (dotted outline); st, stretched muscle (solid outline); Ax, axial probe position (horizontal stripes); Lo, longitudinal probe position (oblique stripes); T0 (left box), T1 (right plot). For example, NPshAxT0 represents the median SWV values among the selected unaffected (non-paretic) muscles of the 14 patients kept in a shortened position with the ultrasound probe in the axial position at T0. Horizontal bars indicate significant comparisons (*p* < 0.01).

Overall, SWV measurements on paretic muscles assessed with a longitudinal positioning of the probe showed statistically significant reduction at T1 vs. T0 both in shortened (*p* = 0.001) and in stretched (*p* = 0.0029) conditions (Figures 5, 6). Conversely, SWV assessments with an axial orientation of the probe did not show a statistically significant reduction between the two time points in stretched (*p* = 0.66) and shortened (*p* = 0.03) conditions (Figure 6).

SWV measurements on non-paretic muscles did not significantly decrease between the two time points (Figure 6).

All SWV measurements on paretic muscles were higher than contralateral non-paretic muscles apart from shortened longitudinal at T1 (*p* = 0.016 for shLoT1, *p* = 0.002 for shAxT0 and shAxT1, and *p* = 0.001 for the other five comparisons) (Figure 6).

Further, SWV measurements obtained in stretched conditions were consistently higher than measures assessed in shortened conditions (*p* = 0.001 for all comparisons) (Figure 6).

**Figure 7:**
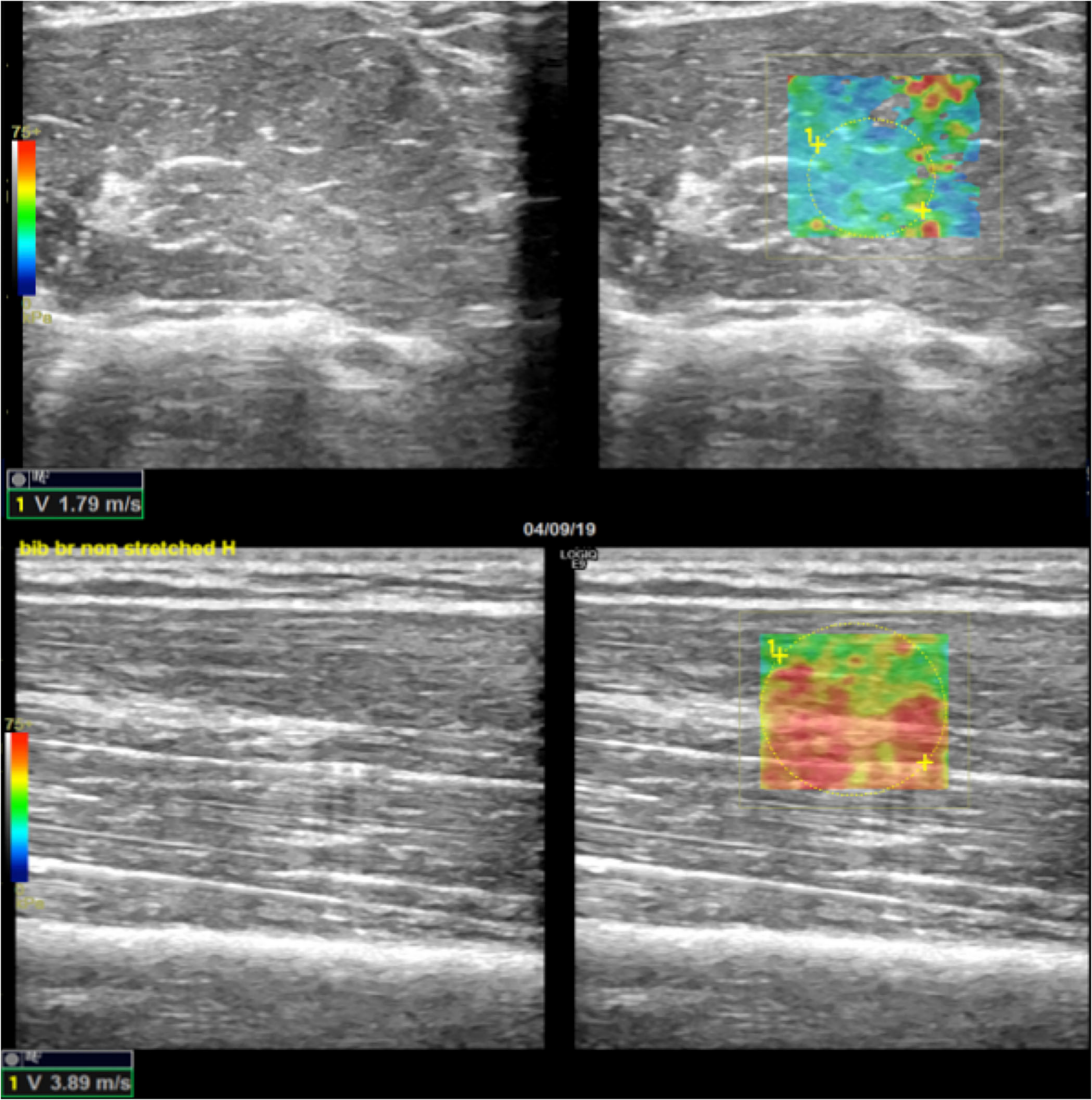
Example of SWE difference between axial and longitudinal assessments. In the upper-right quadrant is illustrated an example of an elastogram (superimposed to the B-mode image) generated by an ROI placed in a non-paretic, shortened biceps brachii muscular belly across the direction of the fibers; in the lower right image, the elastogram generated along the direction of muscular fibers of the same muscle shows higher values expressed both qualitatively with different-colored map and quantitatively in m/s (lower left corners of the images). Images on the upper- and lower-left quadrant are the respective B-mode conventional US image automatically paired on the screen by the SEL software (axial US scan in the upper-left quadrant and longitudinal US scan in the lower-left quadrant).

SWV measurements obtained with a longitudinal positioning of the transducer were also higher than measures performed with the axial orientation of the probe (*p* = 0.001 for all comparisons) (Figures 6, 7).

### Modified Heckmatt scale

**Figure 8:**
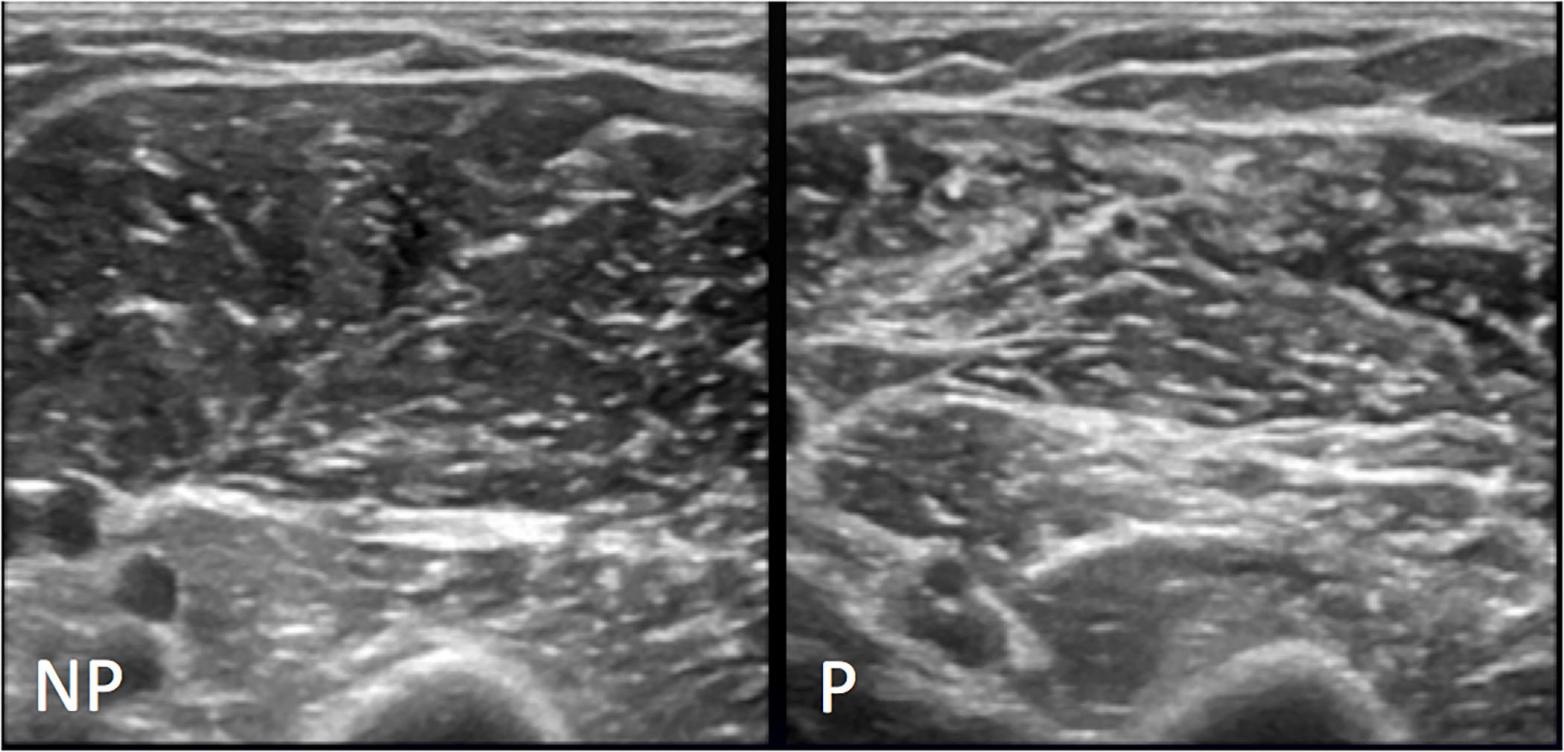
Representative MHS grade II. Conventional US appearance of biceps brachii and brachialis muscles on non-paretic (NP) and paretic side (P) following stroke, as assessed with the axial US scans: on the left image normal US appearance of muscle with regular echogenicity of fibers and normal muscular fibers—intramuscular septa differentiation; the right image shows volume reduction of muscle and increase in background echogenicity with intramuscular septa more represented and still definable.

During the execution of the conventional B-mode US scan, the MHS was applied for the paretic side (Figure 8). MHS scores [T0 and T1: 2 (2, 3)] did not change between T0 and T1. There was a positive correlation between MHS scores and SWV values at T0 and also at T1, more clear with the muscle in a shortened position (T0: Axial Rho = 0.8, *p* = 0.004; Longitudinal Rho = 0.8, *p* = 0.006 – T1: Axial Rho = 0.8, *p* = 0.004; Longitudinal Rho = 0.8, *p* = 0.006) but to a lesser extent also with muscle stretched (T0: Axial Rho = 0.6, *p* = 0.04; Longitudinal Rho = 0.7, *p* = 0.01 – T1: Axial Rho = 0.6, *p* = 0.04; Longitudinal Rho = 0.8, *p* = 0.008). No correlation emerged between MHS and spastic dystonia ARVs.

### Correlation with stroke age

The time elapsed from the stroke to the baseline evaluation (time after stroke, see Table 1) was positively correlated with MHS (Rho = 0.7, *p* = 0.007) but no correlation emerged with SWV, EMG, and MAS values.

## Discussion

To our knowledge, this is the first attempt to quantitatively assess the structural and neural-mediated components in post-stroke patients with spastic dystonia by sEMG and SWE combined assessment. Two main observations resulted from this study. First, SWE, sEMG, and MAS were all able to detect the effect of BoNT-A on paretic muscles in terms of a significant decrease in their values, as previously demonstrated (26, 27). Second, SWE can differentiate paretic from non-paretic muscles. Following BoNT-A injection, both SWV and EMG measurements decreased because both are affected by reflex hypertonia. Such results were expected and should be related to the fact that SWE estimates muscular stiffness by measuring its viscoelastic behavior; hence, it is unable to properly differentiate stiffness due to muscle fibrosis or muscle activity (two simultaneous phenomena which sum up in patients with muscle hypertonia due to upper motor neuron syndrome) (29).

Regarding SWE, a significant decrease at T1 was more evident for measurements obtained with a longitudinal orientation of the probe with respect to the direction of muscular fibers. Such findings reflect the physical properties of skeletal muscle and are comparable with results from published studies (12, 17, 23, 27, 30). Because of the well-known physical assumption that skeletal muscle is an anisotropic (orthotropic), non-linearly viscoelastic, and deformable tissue, shear waves generated into such tissue propagate in different ways with respect to the orientation of muscular fibers. It may be argued that shear waves which propagate on a transverse axis with respect to the direction of the muscular fibers, encounter a greater number of tissue interfaces, resulting in more heterogeneous measurements. Conversely, shear waves that propagate along the direction of the muscular fibers could generate more homogeneous measurements (29). Further, orthotropic physical properties of skeletal muscle are also responsible for the fact that shear waves travel faster along the direction of the fibers than they do when perpendicular to them, which is another point resulted in the present study and already demonstrated by previous papers (12, 15, 31, 32).

Statistically significant differences emerged between velocities detected on paretic muscles and respective contralateral ones, in particular, SWV from paretic muscles was consistently higher. This is also an expected finding since stiffness is increased in paretic muscles compared to the contralateral unaffected side because of higher fibrosis (33) and the presence of EMG activity in a neutral or shortened position (spastic dystonia) and while kept stretched (static phase of spasticity or spastic dystonia occurring in flexor muscles). Furthermore, the greater reduction of SWV detected at T1 in muscles in the stretched condition in comparison to the shortened position was an expected finding due to the exclusive effect of BoNT-A on reflex hypertonia. Conversely, SWV in non-paretic muscles did not consistently change between the two time points T0-T1: this crucial finding indirectly reinforces data resulting from paretic muscles. In 2015, Kesikburun found that SWV was increased in the gastrocnemius muscle on the affected side compared to the unaffected side in subjects with unilateral stroke (34). Some other authors (16, 21, 35) did not show a significant difference between the paretic and non-paretic limbs, but they showed a difference between limbs and control groups supporting the fact that hypertonic muscles tend to have greater SWV than non-hypertonic ones.

Recruitment of patients with quite different time intervals between the stroke and the current assessment (1–15 years) allowed the evaluation of a wide spectrum of muscle changes, considering that muscle changes, particularly the fibrous-fatty substitution of muscle fibers, increase with time. A direct correlation between stroke age and SWV values has been demonstrated in three studies (19, 22, 36). The lack of correlation in this study can be probably attributed to the small sample size, considering also many other nuisance variables that could have influenced muscle stiffness increase after the stroke event, such as the severity of the paresis, the amount and efficacy of rehabilitative treatments, how long the limb remained immobilized, and so on.

Conversely, a good correlation emerged between time since stroke and MHS values, confirming that the structural changes occurring to paretic muscles over time can be detectable with the conventional US. As expected, MHS scores did not change between T0 and T1, confirming that BoNT-A does not affect structural fibrotic changes of paretic muscle, at least after a single treatment. Most importantly, MHS values showed a positive correlation with SWV measurements while the muscle was in the shortened position, but not with spastic dystonia ARV. This finding supports SWE as a useful method to estimate muscle fibrosis independently of the effect of spastic dystonia. It is important to underline that SWE measurement of muscle stiffness cannot tell apart neural-mediated and not neural-mediated muscle hypertonia.

Therefore, as reported in previous studies (17, 24), SWE is sensitive enough to detect stiffness reduction following BoNT-A treatment, but, differently from EMG assessment, cannot demonstrate that SWV reduction is due to decreased muscle activity. Conversely, sEMG is unable to provide information about muscle fibrosis, but when sEMG activity is very limited, as in spastic muscles kept in a shortened position, SWE can provide a measurement of stiffness due almost completely to intrinsic muscle changes.

This study presents many limitations. Our preliminary findings should be reproduced in a large number of subjects and hypertonic muscles from patients with other conditions determining upper motor neuron lesions (such as multiple sclerosis and spinal cord injuries). SWE measurements were obtained only by a single US-experienced operator. SWE has the same issues as ultrasonography, which is operator-dependent, and, to date, it lacks a methodological standardization (12, 15, 27, 37). Indeed, we tried to reduce measurement variability by providing for each condition a final median SWE value resulting from 10 SWE measurements over the entire muscular volume. Even if several studies report good reliability of SWE in the assessment of skeletal muscle stiffness (12–14), given the technical difficulties, SWE requires being properly performed, and less experienced examiners could not achieve the same results. Further, all SWE measurements were conducted with a single US equipment: this could have been a limitationparticularly considering that several manufacturers had implemented their algorithms and post-processing calculation to assess mechanical properties quantifying the shear wave speed.

Electromyographic activity was measured at rest with the examined jointly in a neutral position (spastic dystonia) and while being stretched (dynamic stretch reflex), while SWV was calculated with the muscle in a shortened (not necessarily neutral) and while kept stretched (not during the dynamic phase of the stretching movement). It is possible that the ARV values obtained with the limb in a neutral position could be slightly higher than those obtained with the examined muscle in a shortened position; similarly, the ARV values recorded during the dynamic phase are likely higher than that appearing while the muscle is kept stretched. Furthermore, since EMG and SWE assessments have been performed in different sessions and environmental conditions, we cannot be sure that patients reached the same level of muscle relaxation/contraction during the two measures. Finally, the number of patients enrolled in the study was relatively small. Therefore, future studies assessing different muscle groups in a larger population with post-stroke muscle hypertonia, using multiple US scanners with standardized techniques, are warranted.

In conclusion, our study confirms that SWE is capable of detecting muscle stiffness related to spastic dystonia and the effect of botulinum toxin treatment. The longitudinal probe position for SWE assessment on spastic muscles appears the most suitable to detect stiffness changes. Most interestingly, we showed that muscle hyperechogenicity measured with the MHS positively correlated with SWE in a shortened muscle independently of the contribution of neural-mediated components.

## Data availability statement

The raw data supporting the conclusions of this article will be made available by the authors, without undue reservation.

## Ethics statement

The studies involving human participants were reviewed and approved by Comitato Etico Regionale della Liguria. The patients/participants provided their written informed consent to participate in this study.

## Author contributions

WC performed the electromyographic recording and drafted the manuscript. AC performed the ultrasonographic acquisitions and drafted the manuscript. LPu analyzed electromyographic data. LPr and RP performed the electromyographic recording and collected all data. LMo, LB, GT, and CT critically revised the work for important intellectual content. LMa conceived and designed the study, performed statistical analyses, and drafted the manuscript. All authors approved the final version.

## Conflict of interest

The authors declare that the research was conducted in the absence of any commercial or financial relationships that could be construed as a potential conflict of interest.

## Publisher's note

All claims expressed in this article are solely those of the authors and do not necessarily represent those of their affiliated organizations, or those of the publisher, the editors and the reviewers. Any product that may be evaluated in this article, or claim that may be made by its manufacturer, is not guaranteed or endorsed by the publisher.

## References

[1] Watkins CL Leathley MJ Gregson JM Moore AP Smith TL Sharma AK. Prevalence of spasticity post stroke. Clin Rehabil. (2002) 16:515–22. 10.1191/0269215502cr512oa 12194622

[2] Trompetto C Marinelli L Mori L Pelosin E Currà A Molfetta L. Pathophysiology of spasticity: implications for neurorehabilitation. Biomed Res Int. (2014) 2014:354906. 10.1155/2014/354906 25530960 PMC4229996

[3] Trompetto C Marinelli L Puce L Mori L Serrati C Fattapposta F. “Spastic dystonia” or “Inability to voluntary silence EMG activity”? Time for clarifying the nomenclature. Clin Neurophysiol. (2019) 130:1076–7. 10.1016/j.clinph.2019.03.009 30952436

[4] Trompetto C Currà A Puce L Mori L Serrati C Fattapposta F. Spastic dystonia in stroke subjects: prevalence and features of the neglected phenomenon of the upper motor neuron syndrome. Clin Neurophysiol. (2019) 130:521–7. 10.1016/j.clinph.2019.01.012 30776732

[5] Trompetto C Currà A Puce L Mori L Pallecchi I Gazzola P. Ghost spasticity in multiple sclerosis. J Electromyogr Kinesiol. (2020) 51:102408. 10.1016/j.jelekin.2020.102408 32120056

[6] Puce L Currà A Marinelli L Mori L Capello E Di Giovanni R. Spasticity, spastic dystonia, and static stretch reflex in hypertonic muscles of patients with multiple sclerosis. Clin Neurophysiol Pract. (2021) 6:194–202. 10.1016/j.cnp.2021.05.002 34278056 PMC8263531

[7] Meseguer-Henarejos A-B Sánchez-Meca J López-Pina J-A Carles-Hernández R. Inter- and intra-rater reliability of the modified Ashworth scale: a systematic review and meta-analysis. Eur J Phys Rehabil Med. (2018) 54:576–90. 10.23736/S1973-9087.17.04796-7 28901119

[8] Zúñiga LDO López CAG González ER. Ultrasound elastography in the assessment of the stiffness of spastic muscles: a systematic review. Ultrasound Med Biol. (2021) 47:1448–64. 10.1016/j.ultrasmedbio.2021.01.031 33707090

[9] Klauser AS Miyamoto H Bellmann-Weiler R Feuchtner GM Wick MC Jaschke WR. Sonoelastography: musculoskeletal applications. Radiology. (2014) 272:622–33. 10.1148/radiol.14121765 25153273

[10] Bamber J Cosgrove D Dietrich CF Fromageau J Bojunga J Calliada F. EFSUMB guidelines and recommendations on the clinical use of ultrasound elastography. Part 1: Basic principles and technology. Ultraschall Med. (2013) 34:169–84. 10.1055/s-0033-1335205 23558397

[11] Ryu J Jeong WK. Current status of musculoskeletal application of shear wave elastography. Ultrasonography. (2017) 36:185–97. 10.14366/usg.16053 28292005 PMC5494870

[12] Creze M Nordez A Soubeyrand M Rocher L Maître X Bellin M-F. Shear wave sonoelastography of skeletal muscle: basic principles, biomechanical concepts, clinical applications, and future perspectives. Skeletal Radiol. (2018) 47:457–71. 10.1007/s00256-017-2843-y 29224123

[13] Phan A Lee J Gao J. Ultrasound shear wave elastography in assessment of skeletal muscle stiffness in senior volunteers. Clin Imaging. (2019) 58:22–6. 10.1016/j.clinimag.2019.06.006 31228827

[14] Šarabon N Kozinc Ž Podrekar N. Using shear-wave elastography in skeletal muscle: a repeatability and reproducibility study on biceps femoris muscle. PLoS ONE. (2019) 14:e0222008. 10.1371/journal.pone.0222008 31469888 PMC6716782

[15] Eby S Zhao H Song P Vareberg BJ Kinnick R Greenleaf JF. Quantitative evaluation of passive muscle stiffness in chronic stroke. Am J Phys Med Rehabil. (2016) 95:899–910. 10.1097/PHM.0000000000000516 27149584 PMC5097021

[16] Gao J He W Du L-J Chen J Park D Wells M. Quantitative ultrasound imaging to assess the biceps brachii muscle in chronic post-stroke spasticity: preliminary observation. Ultrasound Med Biol. (2018) 44:1931–40. 10.1016/j.ultrasmedbio.2017.12.012 29398131

[17] Gao J Rubin JM Chen J O'Dell M. Ultrasound elastography to assess botulinum toxin A treatment for post-stroke spasticity: a feasibility study. Ultrasound Med Biol. (2019) 45:1094–102. 10.1016/j.ultrasmedbio.2018.10.034 30898386

[18] Jakubowski KL Terman A Santana RVC Lee SSM. Passive material properties of stroke-impaired plantarflexor and dorsiflexor muscles. Clin Biomech (Bristol, Avon). (2017) 49:48–55. 10.1016/j.clinbiomech.2017.08.009 28866442 PMC5681874

[19] Lee SSM Jakubowski KL Spear SC Rymer WZ. Muscle material properties in passive and active stroke-impaired muscle. J Biomech. (2019) 83:197–204. 10.1016/j.jbiomech.2018.11.043 30551919

[20] Le Sant G Nordez A Hug F Andrade R Lecharte T McNair PJ Gross R. Effects of stroke injury on the shear modulus of the lower leg muscle during passive dorsiflexion. J Appl Physiol (1985). (2019) 126:11–22. 10.1152/japplphysiol.00968.2017 30236050

[21] Mathevon L Michel F Aubry S Testa R Lapole T Arnaudeau LF. Two-dimensional and shear wave elastography ultrasound: A reliable method to analyse spastic muscles? Muscle Nerve. (2018) 57:222–8. 10.1002/mus.25716 28561920

[22] Wu C-H Ho Y-C Hsiao M-Y Chen W-S Wang T-G. Evaluation of post-stroke spastic muscle stiffness using shear wave ultrasound elastography. Ultrasound Med Biol. (2017) 43:1105–11. 10.1016/j.ultrasmedbio.2016.12.008 28285729

[23] Leng Y Wang Z Bian R Lo WLA Xie X Wang R. Alterations of elastic property of spastic muscle with its joint resistance evaluated from shear wave elastography and biomechanical model. Front Neurol. (2019) 10:736. 10.3389/fneur.2019.00736 31354610 PMC6635717

[24] Cao J Xiao Y Qiu W Zhang Y Dou Z Ren J. Reliability and diagnostic accuracy of corrected slack angle derived from 2D-SWE in quantitating muscle spasticity of stroke patients. J NeuroEng Rehabil. (2022) 19:15. 10.1186/s12984-022-00995-8 35120556 PMC8817514

[25] Hermens HJ. European recommendations for surface ElectroMyoGraphy: results of the SENIAM project. In: BV (eds) *Roessingh Research and Development*. Enschede: Roessingh Research and Development (1999). 122 p.

[26] Marinelli L Trompetto C Mori L Vigo G Traverso E Colombano F. Manual linear movements to assess spasticity in a clinical setting. PLoS ONE. (2013) 8:e53627. 10.1371/journal.pone.0053627 23335966 PMC3546077

[27] Lehoux M-C Sobczak S Cloutier F Charest S Bertrand-Grenier A. Shear wave elastography potential to characterize spastic muscles in stroke survivors: Literature review. Clin Biomech (Bristol, Avon). (2020) 72:84–93. 10.1016/j.clinbiomech.2019.11.025 31846849

[28] Moreta MC Fleet A Reebye R McKernan G Berger M Farag J. Reliability and validity of the modified heckmatt scale in evaluating muscle changes with ultrasound in spasticity. Arch Rehabil Res Clin Transl. (2020) 2:100071. 10.1016/j.arrct.2020.100071 33543098 PMC7853393

[29] Miller T Ying M Sau Lan Tsang C Huang M Pang MYC. Reliability and validity of ultrasound elastography for evaluating muscle stiffness in neurological populations: a systematic review and meta-analysis. Physical Therapy. (2021) 101:pzaa188. 10.1093/ptj/pzaa188 33508855

[30] Gennisson J-L Deffieux T Macé E Montaldo G Fink M Tanter M. Viscoelastic and anisotropic mechanical properties of in vivo muscle tissue assessed by supersonic shear imaging. Ultrasound Med Biol. (2010) 36:789–801. 10.1016/j.ultrasmedbio.2010.02.013 20420970

[31] Dorado Cortez C Hermitte L Ramain A Mesmann C Lefort T Pialat JB. Ultrasound shear wave velocity in skeletal muscle: a reproducibility study. Diagn Interv Imaging. (2016) 97:71–9. 10.1016/j.diii.2015.05.010 26119864

[32] Miyamoto N Hirata K Kanehisa H Yoshitake Y. Validity of measurement of shear modulus by ultrasound shear wave elastography in human pennate muscle. PLoS ONE. (2015) 10:e0124311. 10.1371/journal.pone.0124311 25853777 PMC4390150

[33] Liu J Pan H Bao Y Zhao Y Huang L Zhan W. The value of real-time shear wave elastography before and after rehabilitation of upper limb spasm in stroke patients. Biomed Res Int. (2020) 2020:1–7. 10.1155/2020/6472456 32923483 PMC7453236

[34] Kesikburun S Yaşar E Adigüzel E Güzelküçük Ü Alaca R Tan AK. Assessment of spasticity with sonoelastography following stroke: a feasibility study. PM&R. (2015) 7:1254–60. 10.1016/j.pmrj.2015.05.019 26032348

[35] Rasool G Wang AB Rymer WZ Lee SSM. Shear waves reveal viscoelastic changes in skeletal muscles after hemispheric stroke. IEEE Trans Neural Syst Rehabil Eng. (2018) 26:2006–14. 10.1109/TNSRE.2018.2870155 30334740 PMC6471515

[36] Lee SSM Spear S Rymer WZ. Quantifying changes in material properties of stroke-impaired muscle. Clin Biomech. (2015) 30:269–75. 10.1016/j.clinbiomech.2015.01.004 25638688 PMC7057856

[37] Shin HJ Kim M-J Kim HY Roh YH Lee M-J. Comparison of shear wave velocities on ultrasound elastography between different machines, transducers, and acquisition depths: a phantom study. Eur Radiol. (2016) 26:3361–7. 10.1007/s00330-016-4212-y 26815368

